# A randomized clinical study on the efficacy of vonoprazan combined with amoxicillin duo regimen for the eradication of *Helicobacter pylori*

**DOI:** 10.1097/MD.0000000000035610

**Published:** 2023-10-13

**Authors:** Faming Yang, Baiyang Yu, Lang Qin, Xiaorong Dai

**Affiliations:** a Department of Gastroenterology, Taixing Clinical College of Bengbu Medical College, Taixing, China; b Department of Neurology, Taixing Clinical College of Bengbu Medical College, Taixing, China; c Department of Radiotherapy, Taixing Clinical College of Bengbu Medical College, Taixing, China.

**Keywords:** amoxicillin, dual therapy, *Helicobacter pylori*, time efficacy comparison, vonoprazan

## Abstract

**Background:**

*Helicobacter pylori (H pylori*) can cause gastritis, peptic ulcers, gastric cancer, and many other gastrointestinal diseases. The 14-day neo-dual therapy for *H pylori* is considered by most countries to have good eradication rates, while the 7- and 10-day studies have been more widely explored, however, we find that their results are different. The applicability of the shorter and less expensive 10-day neo-dual therapy to our country has not yet been confirmed.

**Methods:**

The patients were divided into 3 groups of 200 each by randomization method. Group A: patients received vonoprazan 20 mg, bid + amoxicillin(1 g), tid, for 14 days. Group B: vonoprazan (20 mg) bid + amoxicillin (1 g) tid, duration of treatment is 10 days, group C: rabeprazole (20 mg) bid + bismuth potassium citrate tablets/tinidazole tablets/clarithromycin tablets, combined package (4.2 g), bid, duration of treatment 14 days. The main comparisons were *H pylori* eradication rate, adverse drug reaction profile and cost—effect ratio in each group.

**Results:**

The eradication rates of groups A, B, and C were 92.5%, 91.6%, and 80.1%, respectively. There was no significant difference in the eradication rates of groups A and B (*P* > .05), groups A and B had statistically significantly better eradication rates than group C (*P* < .05). The incidence of adverse reactions in groups A, B, and C was 9.5%, 8.5%, and 17.0%, respectively. There was no difference in the incidence of adverse reactions between A and B: (*P* > .05), The incidence of adverse reactions was statistically significantly lower in groups A and B than in group C (*P* < .05). Logistic regression analysis showed nonsmokers had a higher eradication rate (OR 2.587, 95% CI: 1.377–4.859, *P* = .003), and taller patients were more likely to have successful eradication (OR 1.052, 95% CI: 1.008–1.097, *P* = .020). Group B had the lowest cost-benefit analysis results.

**Conclusion:**

Group B had an acceptable eradication rate, the lowest incidence of adverse effects, and the lowest cost analysis. Eradication is more likely to be successful in patients who do not smoke and in those who are taller.

## 1. Introduction

After the discovery of *Helicobacter pylori (H pylori*) by Marshall and Warren in the early 1980s, it has served as the focus of research on digestive diseases.^[1]^ Currently, about half of the world’s population is infected with *H pylori*, and according to statistics, the co-infection rate of *H pylori* in mainland China is 44.2% since 1990 to 2019, with an estimated 600 million people infected with *H pylori*.^[2]^ It can also lead to malignant as well as nonmalignant diseases such as gastritis, peptic ulcer, and gastric cancer.^[3,4]^ The eradication of *H pylori* has been a challenge for us and requires constant innovation, from the initial diphtheria, to triple therapy to non-bismuth quadruple to bismuth quadruple, and the *H pylori* eradication rate has been increasing,^[5–7]^ however, it was found in clinical work that the eradication rate of the currently recommended first-line treatment containing bismuth has dropped to <90% and does not yield a satisfactory therapeutic result.^[8]^ At the same time, numerous scholars believe that the use of 2 or 3 or more antibiotics increases resistance, with clarithromycin having the highest resistance and amoxicillin having the lowest resistance, 93.72% and 0.21%, respectively.^[9]^ Therefore, there is an urgent need to break this dilemma and find a regimen with higher eradication rate, better compliance, and higher safety to help the clinic in effective treatment and bring health to patients. Currently, a new acid inhibitor, vonoprazan, is being used in China and abroad as a potassium competitive acid blocker, which acts as a drug prototype without acid activation and has a faster onset of action than traditional proton pump inhibitors or H2 receptor blockers, with a stronger effect of raising pH and a longer half-life.^[10,11]^ And a reasonable, efficient and safe regimen with vonoprazan combined with amoxicillin is still being explored, and mainstream studies have found that 7-day or 10-day therapy does not achieve satisfactory eradication, and similar clinical trials are less available in China, and further data support and protocol optimization are needed to advance clinical treatment in China.^[12]^ The aim of this paper is to study the eradication rate, adverse effects and its cost-effectiveness analysis of 10 days of treatment with vonoprazan in combination with amoxicillin, and to analyze the factors affecting the eradication rate, so that it can be better applied in the clinic to promote the advancement of treatment modalities.

## 2. Methods

### 2.1. Patient selection

This is a randomized controlled study. A total of 600 *H pylori*-positive patients who had not received previous *H pylori* eradication therapy or had previously eradicated *H pylori* but had not undergone eradication therapy within the past 6 months were randomized into 3 groups in a 1:1:1 ratio. The study was reviewed and approved by the Ethics Committee of Taixing People’s Hospital (Ethics Approval No. YJ2022009), the clinical trial registration number: NCT05469685, and all patients obtained the consent of the patients and their families and signed an informed consent form before receiving treatment in this program. Inclusion criteria: All of the following criteria were met to be enrolled in this trial: (1) age 18 to 80 years; (2) patients with a confirmed (+) positive diagnosis of *H pylori*; (3) patients who have not previously received *H pylori* eradication therapy or who have failed prior eradication but have not received eradication therapy within 6 months; (4) patients who voluntarily join this trial and sign the informed consent form. Exclusion criteria: patients who meet one of the following criteria will not be enrolled in this trial: (1) hypersensitivity to the study drug (penicillin allergy, etc); (2) patients with peptic ulcers; (3) patients who have received *H pylori* eradication therapy within 6 months; (4) use of antibiotics, bismuth for 4 weeks prior to initiation of study treatment and histamine H2 receptor antagonists or PPIs for the first 2 weeks; (5) use of adrenocorticosteroids, NSAIDs, or anticoagulants; (6) history of esophageal or gastric surgery; (7) pregnant or lactating women; (8) serious concomitant diseases, such as liver disease, cardiovascular disease, lung disease, kidney disease; (9) alcohol abuse. (10) Excluding gastric mucosa-associated lymphoid tissue lymphoma, malignant neoplastic disease. Termination criteria and exclusion criteria: (1) patients voluntarily withdraw from the trial at any time and for any reason; (2) protocol violation capable of affecting outcome evaluation; (3) discontinuation of the trial by the investigator for safety reasons; (4) serious adverse events; (5) medical or ethical reasons affecting the continuation of the study. Subjects in group A: patients received vonoprazan (Walker) 20 mg, bid + amoxicillin (1 g), tid, for 14 days, group B: vonoprazan (20 mg) bid + amoxicillin (1 g), tid, duration of treatment is 10 days, group C: rabeprazole (20 mg) bid + bismuth potassium citrate tablets/tinidazole tablets/clarithromycin tablets, combined package (4.2 g), bid, duration of treatment 14 days. During the eradication treatment period of groups A, B, and C, respectively, all subjects received instructions and were asked to record their adverse reactions and compliance with the medication. On days 10 and 14 of drug administration, the investigators conducted follow-up telephone and microphone calls to determine adverse effects and compliance. In addition, C13 urea breath test/C14 urea breath test urea breath test was performed within 4 to 6 weeks after the end of treatment to assess the treatment results, and a negative result was considered as successful eradication treatment, while a positive result was considered as failure and were observed. Variables included general information such as age, sex, height, weight, smoking, alcohol consumption, marital status, place of residence, upper gastrointestinal symptoms, upper gastrointestinal diseases, duration of infection, whether a family member was infected or not, educational qualifications, and number of people who shared a meal, and adverse reactions such as nausea, abdominal pain, diarrhea, dizziness, palpitations, and fatigue. Finally, the cost effectiveness was assessed. Cost-effectiveness analysis (time comparison) to calculate the cost-effectiveness ratio, the purpose of the cost-effectiveness analysis is to explore treatment options that are less costly in achieving a given treatment effect. Cost-effectiveness ratio = total cost of 1 course of *H pylori* treatment regimen/*H pylori* eradication rate.

#### 2.1.1. Recording.

Suspected adverse events related to the study drug identified by the investigator that occurred in the subject during the study observation period will be recorded on the CRF’s adverse event form. And the investigator will determine the relevance of the adverse event to the product. The investigator will use the following definitions to assess the relationship between the adverse event and the investigational product: likely to be related: there is a strong temporal relationship between the adverse event and the investigational product, or reuse caused the adverse event, while being unlikely or significantly less likely to be of other etiology. Probably related: there is a strong temporal relationship between the adverse event and the investigational product, and there may be a similar or lesser correlation between another cause and the adverse event. Likely unrelated: There is a small or no temporal relationship between the adverse event and the investigational product and/or there is another more likely etiology. Unrelated: the adverse event was due to an underlying or concomitant disease or drug effect and was unrelated to the investigational product (e.g., no temporal relationship to the investigational product or the presence of another more likely etiology). It is the responsibility of the investigator to notify the Ethics Committee immediately in the event of death or serious injury to the subject. The investigator should keep on file all correspondence with the ethics committee regarding the evaluation of the event.

### 2.2. Statistical methods

Full analysis set: is the analysis of the efficacy of all cases that were randomized and grouped and applied at least once according to the intentionality analysis (ITT) principle. For cases where the full course of treatment was not observed, data from the last observation could be carried forward to the final study outcome. Follow scenario set analysis (PP): efficacy analysis was performed for all cases that met the study protocol, had good compliance, did not take prohibited medications during the study period, and completed the required fields in the case report form. No missing data were filled in. Statistical analysis of drug efficacy was performed for both full analysis set and PP. All statistical tests were performed using a two-sided test, and a *P*-value less than or equal to .05 was considered statistically significant for the difference tested. Measures for each visit in the different treatment groups will be statistically described using the mean ± standard deviation. Comparisons will be made with the screening period baseline values, and paired *t*-tests will be used to compare pre- and post-group differences. Changes before and after treatment in the 3 groups will be compared using analysis of variance. Count data for each visit in different treatment groups were statistically described using frequency (composition ratio). Changes before and after treatment in all 3 groups were examined by chi-square test or nonparametric test. Logistic regression analysis was used to analyze the effect of patients’ general information on eradication rates green. *P*-values less than or equal to .05 were considered statistically significant for the difference of the test.

## 3. Results

### 3.1. General statistics of patients

Table 1 summarizes the baseline characteristics of the patients. A total of 600 patients were enrolled in our study, including 3 groups, A, B, and C, with 200 patients in each group. There were 95 males and 105 females in group A patients, the average age was (48.70 ± 13.4) years old, the average height was (164.00 ± 9.1) centimeters, and the average weight was (61.22 ± 11.2) kilograms. There were 35 cases with junior high school education or below, 115 cases with junior high school or high school education, and 50 cases with college education or above. The number of patients who ate ≥ 3 meals was 134, the number of married patients was 163, the number of patients who never smoked was 167, and the number of patients who never drank alcohol was 154. The number of patients with abdominal distension was 40, diarrhea was 30, abdominal pain was 52, abdominal discomfort was 35, acid reflux was 23, and other symptoms were 10. Thirty-one patients suffered from erosive gastritis, 13 suffered from peptic ulcer, 8 suffered from atrophic gastritis, 25 suffered from reflux esophagitis, 17 suffered from gastric polyps, and 8 suffered from other symptoms. polyps, and 8 patients had other diseases. One hundred fifty-seven *H pylori* infection was diagnosed within 1 week in 157 patients, within 1 month in 24 patients, and within 1 year or more in 19 patients. One hundred seventy-eight patients had family members who were not tested for *H pylori*, 17 patients had family members who were apparently not infected with *H pylori*, and 5 patients had family members who were apparently infected with *H pylori*.

**Table 1:** Demographics of patients.

Variables	Group A	Group B	Group C	Overall
	N = 200	N = 200	N = 200	N = 600
Age (years) (mean ± SD)	48.70 ± 13.4	48.92 ± 11.9	46.01 ± 11.7	
*Sex*				
Male	95 (47.5%)	104 (52%)	98 (49%)	297 (49.5%)
Female	105 (52.5%)	96 (48%)	102 (51%)	303 (50.5%)
Height (cm) (mean ± SD)	164.00 ± 9.1	164.32 ± 8.7	170.07 ± 5.5	
Weight (kg) (mean ± SD)	61.22 ± 11.2	63.63 ± 7.7	63.32 ± 11.4	
*Education status*				
Junior high school and below	35 (17.5%)	49 (24.5%)	48 (24%)	132 (22%)
Secondary and high school	115 (57.5%)	118 (59%)	85 (42.5%)	318 (53%)
College and above	50 (25%)	33 (16.5%)	67 (33.5%)	150 (25%)
*Number of patients eating together*				
<3	66 (33%)	59 (29.5%)	73 (36.5%)	198 (33%)
≥3	134 (67%)	141 (70.5)	127 (63.5%)	402 (67%)
*Marital status*				
Single	37 (18.5%)	24 (12%)	31 (15.5%)	91 (15.3%)
Married	163 (81.5%)	176 (88%)	169 (84.5%)	508 (84.7%)
*Smoking*				
Yes	33 (16.5%)	41 (20.5%)	30 (15%)	104 (17.3%)
No	167 (83.5%)	159 (79.5%)	170 (85%)	496 (82.7%)
*Drinking*				
Yes	46 (23%)	29 (14.5%)	42 (21%)	117 (19.5%)
No	154 (77%)	171 (85.5%)	158 (79%)	483 (80.5%)
*Place of residence*				
Municipalities	131 (65.5%)	132 (66%)	141 (70.5%)	404 (67.3%)
Villagers	69 (34.5%)	68 (34%)	59 (29.5%)	196 (32.7%)
*Digestive symptoms*				401 (66.8%)
Abdominal pain	52 (26%)	36 (18%)	34 (17%)	112 (18.7%)
Bloating	40 (20%)	44 (22%)	44 (22%)	128 (21.3%)
Hiccup	30 (15%)	34 (17%)	24 (12%)	88 (14.7%)
Acid reflux	23 (11.5%)	33 (16.5%)	34 (17%)	90 (15%)
Abdominal discomfort	35 (17.5%)	46 (23%)	36 (18%)	117 (19.5%)
Other	10 (5%)	9 (4.5%)	11 (5.5%)	30 (5%)
*History of upper gastrointestinal diseases*				286 (47.7%)
Erosive gastritis	31 (15.5%)	29 (14.5%)	27 (13.5%)	87 (14.5%)
Peptic ulcer	13 (6.5%)	11 (5.5%)	22 (11%)	46 (7.7%)
Atrophic gastritis	8 (4%)	9 (4.5%)	18 (9%)	35 (5.8%)
Reflux esophagitis	25 (12.5%)	27 (13.5%)	18 (9%)	70 (11.7%)
Gastric polyps	17 (8.5)	9 (4.5%)	6 (3%)	32 (5.3%)
Other	8 (4%)	6 (3%)	2 (1%)	16 (2.7%)
*Time of H pylori infection*				
<1week	157 (78.5%)	144 (72%)	151 (75.5%)	452 (75.3%)
<1 year	24 (12%)	37 (18.5%)	32 (16%)	93 (15.5%)
>1 year	19 (9.5%)	19 (9.5%)	17 (8.5%)	55 (9.2%)
*H pylori infection in a family member*				
Unchecked	178 (89%)	175 (87.5%)	177 (88.5%)	530 (88.3%)
No	17 (8.5%)	16 (8%)	13 (6.5%)	46 (7.7%)
Yes	5 (2.5%)	9 (4.5%)	10 (5%)	24 (4%)

*H pylori* = *Helicobacter pylori*.

In group B, there were 104 males and 96 females; the average age of the patients was (48.92 ± 11.9) years old, the average height was (164.32 ± 8.7) centimeters, and the average weight was (63.63 ± 7.7) kilograms. Forty-nine patients had junior high school education or below, 118 patients had middle school and high school education, and 33 patients had college education or above. The number of patients with ≥ 3 meals was 141, the number of married patients was 176, the number of patients who never smoked was 159, and the number of patients who never drank alcohol was 171. The number of patients with abdominal distension was 44, hiccup was 34, abdominal pain was 36, abdominal discomfort was 46, acid reflux was 33 and other symptoms were 9. Twenty-nine patients had erosive gastritis, 11 patients had peptic ulcer, 9 patients had atrophic gastritis, 27 patients had reflux esophagitis, 9 patients had gastric polyps and 6 patients had other diseases. There were 175 patients whose family members were not tested for *H pylori*, 16 patients whose family members were clearly free of *H pylori* infection, and 9 patients whose family members were clearly infected with *H pylori*. 144 patients were diagnosed with *H pylori* infection within 1 week, 37 patients were diagnosed with *H pylori* infection within 1 month, and 19 patients were diagnosed with *H pylori* infection at 1 year or more.

There were 98 males and 102 females in group C. The average age of the patients was 46.01 ± 11.7 years, the average height was (170.07 ± 5.5) centimeters, and the average weight was (63.32 ± 11.4) kilograms. Forty-eight patients had junior high school education or below, 85 patients had secondary school and high school education, and 67 patients had college education or above. The number of patients who dined with ≥ 3 people was 127, 169 were married, 170 never smoked, and 158 never drank alcohol. The number of patients with bloating was 44, hiccup was 24, abdominal pain was 34, abdominal discomfort was 36, acid reflux was 34 and other symptoms were 11. There were 27 patients with erosive gastritis, 22 patients with peptic ulcer, 18 patients with atrophic gastritis, 18 patients with reflux esophagitis, 6 patients with gastric polyps, and 2 patients with other diseases. One hundred fifty-one patients were diagnosed with *H pylori* infection within 1 week, 32 patients within 1 month, and 17 patients more than 1 year. There were 177 patients whose family members were not tested for *H pylori*, 13 whose family members were definitively free of *H pylori*, and 10 whose family members were definitively *H pylori*.

### 3.2. Comparison of *H pylori* eradication rate of patient

As shown in Table 2, in groups A, B, and C, 200 patients were enrolled in each group. One hundred seventy-two patients in group A completed the medication as prescribed, 14 failed and 14 fell off; 174 patients in group B completed the medication as prescribed, 16 failed and 10 fell off; 141 patients in group C completed the medication as prescribed, 35 failed and 24 fell off. We used PP analysis to compare the eradication rates between the groups. Groups A and B no significant difference in efficacy rate (*P* > .05); groups B and C difference in efficacy rate (*P* < .05); groups A and C is difference in efficacy rate (*P* < .05).

**Table 2:** Eradication rates.

Group	A	B	C	*P*-value
Success: (cases)	172	174	141	
Failure: (cases)	14	16	35	
Missed visits: (cases)	14	10	24	
Total (cases)	200	200	200	
PP (%)	92.5	91.6	81.5	
P^*^				.749
P^**^				<.001
P^***^				.002

*Notes*: Adopted PP(%), P* = groups A and B contrast, P** = groups A and C contrast, P*** = groups B and C contrast.

### 3.3. Comparison of the incidence of adverse reactions in patients

As shown in Table 3, We used ITT to analyze the incidence of adverse reactions between the groups. Groups A and B: there was no statistical difference in the incidence of adverse reactions (*P* > .05), and there was a statistical difference in the incidence of adverse reactions between groups B and C (*P* < .05), and a statistical difference in the incidence of adverse reactions between groups A and C (*P* < .05).

**Table 3:** Incidence of adverse reactions in the 3 groups of patients.

Group	A	B	C	*P*-value
Nausea (cases)	9	5	20	
Vomiting (cases)	0	0	1	
Abdominal pain (cases)	3	4	2	
Diarrhea (cases)	1	2	4	
Dizziness (cases)	2	1	1	
Weakness (cases)	2	2	2	
Palpitations (cases)	0	1	2	
Total	9.5%(17/200)	8.5%(15/200)	17% (32/200)	
P^*^				.727
P^**^				.027
P^***^				.011

*Notes*: Adopted ITT(%), P* = groups A and B contrast, P** = groups A and C contrast, P*** = groups B and C contrast.

### 3.4. Cost-effectiveness analysis

The treatment cost of group A was about 305.54, the treatment cost of group B vonoprazan for a period of B was about 226.39, the treatment cost of group C was about 447.32, the cost effect ratio of group A was 3.30 (305.54/92.5), the cost effect ratio of group B was 2.47 (226.39/91.6), and the cost effect ratio of group C was 5.58 (447.32/ 80.1). Collectively, group A has the lowest cost-effect ratio and is better than groups B and C.

### 3.5. Relationship between general patient information and eradication rates

As shown in Table 4, eradication rates were higher among nonsmokers (OR 2.587, 95% CI: 1.377–4.859, *P* = .003), and taller patients were more likely to have successful eradication (OR 1.052, 95% CI: 1.008–1.097, *P* = .020).Secondly, age, gender, weight, alcohol consumption, marital status, place of residence, upper gastrointestinal symptoms, upper gastrointestinal diseases, duration of infection, whether family members were infected, education and number of people sharing a meal did not influence the success of *H pylori* eradication.

**Table 4:** Results of the analysis of factors affecting the eradication rate of *Helicobacter pylori*.

Variant		OR (95% CI)	*P*-value
Constant		0.001	.064
*Sex*			
Males	Reference		
Female		1.015 (0.5841.765)	.958
Age		1.008 (0.983–1.033)	.525
Height		1.052 (1.008–1.097)	.020
Weight		0.983 (0.956–1.011)	.240
*Number of patients eating together*			
<3	Reference		
≥3		0.656 (0.359–1.199)	.171
*Marital status*			
Married	Reference		
Single		1.649 (0.700–3.884)	.253
*Place of residence*			
Municipalities	Reference		
Villagers		1.006 (0.567–1.782)	.985
*Smoking*			
Yes	Reference		
No		2.587 (1.377–4.859)	.003
*Drinking*			
Yes	Reference		
No		0.758 (0.368–1.561)	.453
*Digestive symptoms*			
Yes	Reference		
No		1.014 (0.575–1.789)	.961
*History of upper gastrointestinal disease*			
Yes	Reference		
No		1.312 (0.766–2.246)	.323
*Time of H pylori infection*			
<1 week	Reference		.804
<1 year		1.226 (0.575–2.618)	.598
>1 year		0.866 (0.359–2.091)	.749
*H pylori infection in a family member*			
Unchecked	Reference		.488
No		1.181 (0.190–7.336)	.858
Yes		0.606 (0.198–1.852)	.379

CI = confidence interval, *H pylori* = *Helicobacter pylori*, OR = odds ratio.

## 4. Discussion

According to this study, the eradication rate of vonoprazan combined with high-dose amoxicillin in groups A and B was similar, without statistical difference, and both were better than the current mainstream regimen: quadruple therapy. From the study of adverse reactions, from ITT analysis, there was no significant difference in adverse reactions between groups A and B; There was a statistical difference between groups A and C (*P* < .05), and also between groups B and C (*P* < .05), and the incidence of adverse reactions was lower than that of group C. The incidence of adverse reactions was lower than that of group C, which was safer and more effective. The combination of high dose of vonoprazan and amoxicillin for 10 days was more effective, less expensive to treat and safer.

Meta-analysis showed that 3 doses of amoxicillin per day improved eradication rates compared to the traditional 2 doses per day at the same total amoxicillin dose. The optimal therapeutic dose of amoxicillin is 3 grams per day, and the frequency of 3 doses per day improves the eradication rate.^[13]^ Also, in one of our foreign reports, we found that amoxicillin has the lowest resistance rate and is most suitable for *H pylori* eradication, which can reduce the incidence of eradication failure events due to drug resistance, and amoxicillin also has pH-dependent properties. In addition, the higher the intragastric pH, the more stable the action of amoxicillin and the stronger the bactericidal effect. In addition, a significant proportion of *H pylori* is either not replicating or replicating very slowly at any given time. This is a challenge for antibiotics that need to rely on microbial replication mechanisms to kill the germs (e.g., amoxicillin). This is because amoxicillin only has a bactericidal effect when the pH rises to 6 to 8 and *H pylori* enters division and proliferation. Because *H pylori* does not have β-lactamase, amoxicillin is more beneficial for *H pylori* eradication.^[14,15]^

Vonoprazan is mainly metabolized by the hepatic microsomal enzyme Cytochrome P4503A4 enzymes and is not affected by Cytochrome P450, family 2, subfamily C, polypeptide 19 gene polymorphism, while vonoprazan has a long plasma half-life and remains stable in an acidic environment, so it can stay in the secretory tubules for a long time and can inhibit both resting and activated state proton pumps by binding to H+/K+-ATPase through ionic and hydrogen bonds.^[16]^ The drug is able to rapidly increase intragastric pH, reaching maximum acid suppression on the first day, and the intragastric pH4 was significantly higher than rabeprazole in the whole day on days 1 and 7 of administration.^[17]^ In phase III clinical studies conducted in Europe and the United States, the eradication rates of nonresistant infections with vonoprazan triple therapy and vonoprazan dual therapy were 84.7% and 78.5%, respectively, not inferior to proton pump inhibitors -based eradication rates of *H pylori*.^[18]^ The data show that the overall PP eradication rate for vonoprazan dual or triple combination therapy is >90%,^[19–21]^ providing additional options for optimizing dual combination therapy.^[11,22,23]^

Adverse effects are also an important indicator for assessing the rationality of the treatment program. In the ITT analysis, we observed that there was no statistically significant difference in adverse reactions between groups A and B, a statistically significant difference between groups A and C, and a statistically significant difference between groups B and C. Group B had a better safety profile among the 3 groups.

Literature shows that smoking leads to a further decrease in the pH value in the stomach. Secondly, nicotine also reduces the blood flow to the gastric mucosa, reducing the bactericidal effect of antibiotics. At the same time, it is difficult for smoking patients to quit smoking, which makes it impossible to quit smoking during the treatment period and makes it difficult to eradicate *H pylori*. At the beginning of the experimental design, we failed to break down the number of cigarettes smoked by the patients per day to determine whether the eradication rate increases with the number of cigarettes smoked, and we currently believe that the dose of medication should be increased in patients who smoke in order to improve the eradication rate of *H pylori*. In addition to this, height is also a factor that affects the eradication rate of *H pylori*, with taller patients being more likely to eradicate *H pylori*, but as this mainly refers to the study of the effect of different dosing regimens on the eradication rate the optimal threshold for height could not be better analyzed in this study. We will analyze this in the next trial.^[24,25]^

In recent years, primary and secondary antibiotic resistance of *H pylori* has been increasing year by year. For patients who fail to eradicate *H pylori* our next step is to consider a noninvasive method, that is, collecting stool and then sending it to a specialized institution for laboratory testing to determine which drugs the patient is resistant to and then sterilizing it to improve the eradication rate of *H pylori*. Detection of drug-resistant genotype I antibiotic resistance is currently the main reason for the failure of *H pylori* eradication, and the main determinant of successful *H pylori* eradication is to understand its resistance to antibiotics before treatment; the use of PCR technology to detect drug-resistant genotype has important clinical value in guiding the selection of antibiotics and is superior to traditional bacterial culture and drug sensitivity tests. Although molecular biology techniques are not routinely used to diagnose *H pylori* infection, they are highly sensitive and specific, similar to other methods. “The Maastricht VI/Florence consensus report”^[26]^ on the diagnosis and treatment of *H pylori* mainly mention for antibiotic resistance genetic testing: molecular methods (especially real-time polymerase chain reaction, whole genome sequencing and digital polymerase chain reaction) can detect *H pylori* mutations associated with clarithromycin, levofloxacin, tetracycline and rifampicin resistance, which is important for guiding *H pylori* eradication treatment. With the advantages of noninvasive, simple and convenient sampling, high sensitivity and specificity, fecal *H pylori* molecular testing has become a hot spot for precise diagnostic research and treatment of *H pylori* infection.

In conclusion, there is no significant difference between group B eradication therapy and group A eradication therapy, and it is better than the current mainstream quadruple therapy, and there is no significant difference between group B and group A adverse effects, and it has higher safety than group C, and eradication of *H pylori* is easier in nonsmokers and in taller patients. Therefore, the 10-day treatment of Vonoprazan combined with amoxicillin can be an effective way to eradicate *H pylori* in Jiangsu Taixing.

## Author contributions

**Data curation:** Baiyang Yu.

**Software:** Lang Qin.

**Writing – original draft:** Faming Yang.

**Writing – review & editing:** Xiaorong Dai.
